# Combined clustering and association-rule analysis for hospital costs and length of stay in spontaneous intracerebral hemorrhage hospitalizations

**DOI:** 10.3389/fpubh.2026.1828871

**Published:** 2026-06-19

**Authors:** Qiuping Li, Ying Wang, Suren Rao Sooranna, Lihong Zhao, Liuying Tang

**Affiliations:** 1 Faculty of Nursing, Youjiang Medical University for Nationalities, Baise, China; 2 Department of Metabolism, Digestion and Reproduction, Faculty of Medicine, Imperial College London, London, United Kingdom; 3 Department of Infectious Diseases, Baise People's Hospital, Baise, China

**Keywords:** association-rule mining, DRG/DIP, Gower distance, hospital cost, intracerebral hemorrhage, k-medoids clustering, length of stay, resource utilization

## Abstract

**Introduction:**

Intracerebral hemorrhage (ICH) imposes a substantial burden on inpatient resources, yet hospital costs and length of stay (LOS) are often evaluated as separate endpoints. This study aimed to identify interpretable clinical-administrative strata and recurrent high-utilization care patterns among spontaneous ICH hospitalizations.

**Methods:**

This single-center retrospective cohort study included 1,851 spontaneous ICH hospitalizations from 5 November 2022 to 31 October 2024. Clustering variables were restricted to age, admission source, diagnosis-derived hemorrhage-location category, primary ventricular hemorrhage/intraventricular extension, hypertension-coded status, diabetes, and chronic kidney disease/renal failure. Cost-composition ratios, total cost, LOS, procedures, discharge disposition, departments, and acute in-hospital complications were excluded from cluster formation and reserved for descriptive analyses, adjusted modeling, or association-rule mining. Gower dissimilarities with k-medoids clustering were evaluated across K = 2–8 using elbow analysis, average silhouette coefficient, permutation-based gap statistic, subsampling stability, cluster size, clinical interpretability, and parsimony.

**Results:**

The *K* = 3 solution was retained as the parsimonious working solution, with the highest average silhouette coefficient among *K* = 2-7 (0.440), a 14.7% reduction in within-cluster dissimilarity from *K* = 2, acceptable subsampling stability (mean adjusted Rand index, 0.702), and no small outlier cluster (minimum *n* = 308). The three strata were emergency-presentation (*n* = 450), hypertension-uncoded/lobar-leaning (*n* = 308), and non-emergency hypertension-coded/deep-ICH (*n* = 1,093). Unadjusted total cost and LOS differed across clusters, but these differences were attenuated after adjustment for sex, payment method, admission department, secondary-diagnosis burden, complications, and procedures. High-cost and prolonged-LOS association rules were dominated by tracheostomy-centered combinations involving DVT/PE, CVC/PICC, chronic kidney disease/renal failure, pneumonia, respiratory failure, or major neurosurgery.

**Discussion:**

This pathway-oriented framework organized ICH hospitalizations into interpretable baseline clinical-administrative strata and identified recurrent comorbidity/procedure/complication patterns associated with high utilization. These findings may support risk-aware benchmarking and resource management under diagnosis-related group/diagnosis-intervention packet payment reform, although validation using granular clinical severity data is required.

## 1 Introduction

Intracerebral hemorrhage (ICH) is one of the most devastating forms of stroke and contributes disproportionately to early mortality, disability, and long-term care needs (1, 2). Although ICH accounts for a minority of all strokes, acute management often requires emergency triage, neurocritical monitoring, neurosurgical decision-making, airway support, and prevention or treatment of in-hospital complications. These processes can generate a substantial variation in inpatient costs and length of stay (LOS), creating challenges for hospital management, pathway evaluation, and value-based care assessment (3–5).

Previous studies have identified individual factors associated with higher expenditure or longer hospitalization after ICH, including operative interventions, comorbidities, complications, and intensive care requirements (3–5). However, inpatient resource utilization is rarely driven by isolated factors. Hospitalizations often involve recognizable combinations of presenting characteristics, care settings, comorbidities, procedures, and complications. Data-driven approaches may therefore complement conventional regression models by identifying clinically interpretable strata and recurrent comorbidity/procedure/complication patterns relevant to pathway-based resource management practices.

Interpretable machine-learning and electronic health record (EHR) approaches are increasingly used in stroke research to support transparent risk stratification. Lv et al. developed an interpretable model for 30-day readmission after stroke, demonstrating the value of data-driven modeling that preserves clinically interpretable feature patterns (6). Such work is largely supervised and is often prediction oriented. Unsupervised clustering and association-rule mining offer a complementary descriptive strategy for identifying naturally occurring strata and high-lift co-occurrence patterns within inpatient care.

For administrative resource-utilization research, strata should, whenever possible, be defined using information available before or early during hospitalizations, while costs and LOS should be evaluated as downstream utilization outcomes. Detailed clinical severity measures, such as hematoma volume, the Glasgow coma scale value, ICH score, mechanical ventilation duration, neurological status, and care-limitation decisions, are not always available in hospital information system (HIS) datasets. In this setting, admission route, diagnosis-derived hemorrhage location, ventricular involvement, and chronic comorbidity codes can serve as pragmatic, although incomplete, clinical-administrative signals for pathway review.

Accordingly, this study used baseline clinical-administrative variables to cluster ICH hospitalizations and then examined costs, LOS, procedures, complications, and discharge-related factors as descriptors or outcomes. Specifically, we aimed to identify interpretable clinical-administrative strata among ICH hospitalizations and evaluate the number of clusters using multiple validation criteria. In addition, we examined costs and LOS differences before and after adjustments for available administrative severity and care-process proxies and characterized the comorbidity/procedure/complication co-occurrence patterns associated with high costs and prolonged LOS using association-rule mining. Rather than treating high cost or prolonged LOS as homogeneous endpoints or isolated predictors, this design frame resource was used as a set of baseline strata and recurrent pathway signals while keeping the downstream utilization variables, out of the cluster formation. By linking these components, the framework obtained provides a pathway-oriented resource-utilization analysis that is consistent with a structure-process-outcome view of healthcare evaluations (7) and this is clinically interpretable for diagnosis-related group/diagnosis-intervention packet (DRG/DIP)-oriented hospital management.

## 2 Materials and methods

### 2.1 Study design and population

This retrospective observational study included consecutive hospitalizations with a primary HIS diagnosis compatible with spontaneous ICH at the Affiliated Hospital of Youjiang Medical University for Nationalities, Guangxi, China, between 5 November 2022 and 31 October 2024. The data were extracted from the HIS and inpatient billing module. The hospitalization episode was the unit of analysis. The eligible records were required to have a primary HIS diagnosis indicating spontaneous ICH and non-missing values for variables prespecified for clustering, outcome modeling, and association-rule mining. Records were excluded if they did not meet the spontaneous ICH case definition, including traumatic, subarachnoid, tumor-related, or other non-spontaneous hemorrhage when identifiable from HIS diagnosis fields, or if values were missing for variables required for clustering, outcome modeling, or association-rule mining. No records were excluded based on sex, payment method, procedures, discharge disposition, cost level, or LOS. After exclusions, 1,851 hospitalizations were included. The study protocol was approved by the Ethics Committee of Youjiang Medical University for Nationalities (approval no.: KY2024102863). Since only de-identified retrospective administrative data were used for this study, the committee waived the need for individual informed consent from patients.

### 2.2 Clinical-administrative variable definitions and coding

Clustering variables were selected *a priori* to represent baseline demographic, admission-route, diagnosis-derived, and chronic comorbidity information available before or early during hospitalizations. The input set comprised of age (years), admission source, hemorrhage-location category derived from the primary diagnosis, primary ventricular hemorrhage/intraventricular hemorrhage (IVH) extension, hypertension-coded status, diabetes, and chronic kidney disease (CKD)/renal failure. The variables included in and excluded from the clustering model are summarized in Supplementary Table S1 and variable selection was informed by prior ICH resource-utilization studies (3–5), clinical plausibility, and availability in the administrative dataset.

Admission source was dichotomized as emergency versus non-emergency using the HIS admission-source field. In this study, non-emergency denoted the HIS admission-source category and should not be interpreted as indicating the absence of acute clinical urgency. All binary diagnoses, procedures, and complication indicators were coded as 1 = present and 0 = absent, according to prespecified code/name mappings. Hypertension-coded status was coded as 1 when a hypertension diagnosis code or diagnosis name was recorded and as 0 when it was not recorded. The uncoded category should not be interpreted as confirmed absence of hypertension. Diabetes and CKD/renal failure were coded analogously. The method of payment and the admission department group were treated as nominal covariates. Cluster membership was assigned after clustering as C1, C2, or C3, and high-cost and prolonged-LOS indicators were coded as 1 only when the prespecified upper-quartile threshold was exceeded. A detailed coding summary is provided in Supplementary Table S2.

Primary diagnosis names were mapped into six hemorrhage-location categories: deep, lobar, brainstem, cerebellar, primary intraventricular, and other/unspecified. Deep hemorrhage included basal ganglia, thalamus, external capsule, caudate, and diencephalic labels, whereas lobar hemorrhage included frontal, temporal, parietal, occipital, cortical/subcortical, multilobar, and corpus callosum labels. A separate IVH indicator captured primary ventricular hemorrhage or ventricular extension. The secondary-diagnosis count was defined as the number of non-primary discharge diagnosis entries recorded in the HIS.

The total inpatient cost was recorded in RMB and LOS in days. Cost and LOS were treated as utilization outcomes or descriptive measures. Cost-composition ratios, procedures, discharge department, discharge disposition, and acute in-hospital complications were not used for cluster formation. The procedure indicators included any surgery, major neurosurgery, tracheostomy, feeding tube/gastrostomy, and central venous catheter/peripherally inserted central catheter (CVC/PICC). Acute complication indicators included pneumonia, respiratory failure, urinary tract infection (UTI), deep venous thrombosis/pulmonary embolism (DVT/PE), and sepsis. These procedure and complication indicators were derived from structured HIS procedure records and discharge diagnosis fields using predefined code/name mappings and they were reserved for cohort description, model adjustment, interpretation, or association-rule mining.

### 2.3 Clustering algorithm and selection of K

Because the input set contained mixed numeric, categorical, and binary variables, we calculated the Gower dissimilarities (8). Age was range-scaled within the cohort, and categorical/binary variables were compared using simple matching. K-medoids clustering was applied to the Gower dissimilarity matrix because medoid-based clustering can accommodate arbitrary dissimilarity matrices and they represent each cluster by an observed hospitalization (9). Candidate solutions from *K* = 2 to *K* = 8 were evaluated using a prespecified combination of within-cluster dissimilarity elbow analysis, average silhouette coefficient (10), permutation-based gap statistic with 20 reference datasets generated by independently permuting each clustering variable (11), subsampling stability calculated as the adjusted Rand index (ARI) between full-data clusters and clusters derived from 40 random 70% subsamples (12), cluster size, clinical interpretability, and parsimony. No single validation index was used as the sole selection criterion and instead, the final solution was selected by balancing internal validation, subsampling stability, cluster size, clinical interpretability, and parsimony.

### 2.4 Cluster characterization

Cluster labels were assigned after clustering according to dominant clinical-administrative characteristics. Variables not used in clustering, and these include sex, payment method, admission department group, secondary-diagnosis count, procedures, complications, total cost, and LOS, and they were only used for cluster characterization. Continuous-variable distributions were assessed using histograms, quantile-quantile plots, skewness, and equality-of-variance checks. Approximately symmetric continuous variables were summarized as mean ± SD and compared using one-way ANOVA, after a Welch correction when the homogeneity-of-variance assumption was not satisfied. The skewed continuous variables were summarized as medians [interquartile ranges (IQR)] and compared using the Kruskal–Wallis tests. The categorical variables were summarized as n (%) and compared using Pearson Chi-square tests, with Fisher’s exact tests planned for sparse tables with expected cell counts <5.

### 2.5 Outcome definitions and adjusted models

Outcomes were total inpatient cost, LOS, and prolonged LOS. High cost and prolonged LOS were defined using cohort-specific upper-quartile thresholds: total inpatient cost > RMB 86,600.10 and LOS >25 days. The high-cost indicator was used for descriptive summaries and as a consequent in association-rule mining, whereas continuous total cost was retained as the regression outcome to avoid unnecessary dichotomization. Before model fitting, we confirmed that total cost values were strictly positive and skewed to the right and LOS overdispersion was assessed by comparing the variance with the mean. Total cost was modeled using a gamma generalized linear model (GLM) with a log link because healthcare cost data are positive and typically right-skewed (13). LOS was modeled using negative binomial regression because the LOS is count-like and it is commonly over-dispersed relative to a Poisson distribution (14). Prolonged LOS was modeled using logistic regression because it was a binary outcome. Model coefficients were exponentiated and reported as cost ratios, incidence rate ratios (IRRs), or odds ratios (ORs), respectively, with 95% confidence intervals (CIs). Cluster 3, the largest non-emergency hypertension-coded/deep-ICH stratum, was used as the reference category. Base models were adjusted for sex, payment method, admission department group, and secondary-diagnosis count. Extended models were additionally adjusted for pneumonia, respiratory failure, UTI, DVT/PE, sepsis, any surgery, major neurosurgery, tracheostomy, feeding tube/gastrostomy, and CVC/PICC. Extended models were used to evaluate attenuation after accounting for available administrative severity and care-process proxies; they were not intended to estimate causal mediation. Because cluster membership was derived from age, admission source, hemorrhage location, ventricular involvement, and chronic comorbidity indicators, these clustering input variables were not re-entered as separate covariates in the models which contained cluster indicators to reduce collinearity and over-adjustment.

### 2.6 Association-rule mining

Association-rule mining was performed using the Apriori framework (15) to identify recurrent comorbidity/procedure/complication combinations associated with high utilization. Selected chronic comorbidity indicators (hypertension-coded status, diabetes, and CKD/renal failure), procedure indicators, and acute in-hospital complication indicators were specified only as antecedent items, whereas high cost and prolonged LOS were specified only as consequents. Antecedent itemsets were limited to one or two items to preserve interpretability. Candidate rules required support ≥0.02 and confidence ≥0.60; these thresholds were set *a priori* to retain rules that occurred in approximately 2% or more of hospitalizations while requiring that most episodes with the antecedent also met the outcome definition. Support was defined as the proportion of all hospitalizations containing both the antecedent and consequent itemsets. Confidence was the proportion of hospitalizations with the antecedent itemset that also had the consequent and lift was the observed confidence divided by the marginal probability of the consequent. Rules were ranked by lift, which was interpreted as the strength of co-occurrence beyond the marginal outcome frequency. No redundancy pruning was applied and the overlapping rules were interpreted as related rule families rather than as independent patterns. Association-rule mining was descriptive and did not establish temporality or causation. Visualization followed the rationale that interpretable pathway visualizations can summarize complex care trajectories (16).

### 2.7 Statistical software, assumptions, and reproducibility

Descriptive comparisons and multivariable regression models were performed using SPSSAU version 26.0 (Qingsi Technology, Beijing, China). Data checking, Gower distance calculations, k-medoids clustering, K-validation summaries, association-rule summaries, model-output compilation, and figures were generated from de-identified analysis tables using Python 3.11 (Python Software Foundation, Wilmington, DE, USA) with pandas, NumPy, scikit-learn, scikit-learn-extra, statsmodels, mlxtend, and matplotlib. The clustering inputs, distance metric, K-validation procedure, model families, covariates, Apriori thresholds, rule-ranking criteria, and model-selection checks are reported above and in the Supplementary material to support reproducibility. Stability was evaluated using 40 random 70% subsamples. The rationale, assumptions, and diagnostics for each major analysis are summarized in Supplementary Table S3. All hypothesis tests were two-sided, and *p* < 0.05 was considered statistically significant. Since cluster comparisons and rule mining were exploratory and descriptive, their *p* values and rule rankings were interpreted without multiplicity-adjusted confirmatory claims.

## 3 Results

### 3.1 Cohort characteristics

The final cohort comprised 1,851 hospitalization episodes. The mean age was 58.2 ± 11.9 years, and 1,366 episodes involved male patients (73.8%). Emergency admissions occurred in 492 hospitalizations (26.6%). The median total inpatient cost was RMB 27,452.99 [IQR 11,593.06–86,600.10], and median LOS was 14 days [IQR 6–25].

### 3.2 K selection and cluster solution

The *K* = 3 clinical-administrative solution identified three clusters: Cluster 1, the emergency-presentation stratum (*n* = 450, 24.3%); Cluster 2, the hypertension-uncoded/lobar-leaning stratum (*n* = 308, 16.6%); and Cluster 3, the non-emergency hypertension-coded/deep-ICH stratum (*n* = 1,093, 59.0%). Hypertension-uncoded denotes the absence of a hypertension diagnosis code in the HIS and should not be interpreted as confirmed absence of hypertension.

With respect to *K* = 2–8, the *K* = 3 solution provided the most balanced trade-off across internal validation, subsampling stability, cluster size, clinical interpretability, and parsimony. It had the highest average silhouette coefficient among *K* = 2–7 (0.440), a 14.7% reduction in within-cluster dissimilarity from *K* = 2, acceptable subsampling stability (mean ARI, 0.702), and no small outlier cluster (minimum *n* = 308). *K* = 2 had higher subsampling stability but was clinically too coarse, whereas *K* = 8 had a similar average silhouette coefficient (0.436) but over-fragmented the cohort, including a 68-case cluster. Therefore, *K* = 3 was interpreted as a parsimonious working solution rather than as a universally optimal cluster number. All the validation metrics, cluster-specific silhouette values, and validation plots are provided in Supplementary Tables S4, S5 and Supplementary Figure S1. Table 1 summarizes the baseline and severity-proxy characteristics of the overall cohort and the three clinical-administrative clusters.

**Table 1:** Baseline and severity-proxy characteristics of the overall cohort and clinical-administrative clusters.

Variable	Overall	C1 emergency-presentation	C2 hypertension-uncoded/lobar-leaning	C3 non-emergency hypertension-coded/deep-ICH	*p* value
*n* (%)	1,851 (100.0)	450 (24.3)	308 (16.6)	1,093 (59.0)	—
Age, years, mean ± SD	58.2 ± 11.9	59.1 ± 11.5	56.5 ± 13.6	58.3 ± 11.5	0.025
Male sex, *n* (%)	1,366 (73.8)	332 (73.8)	226 (73.4)	808 (73.9)	0.981
Emergency admission, *n* (%)	492 (26.6)	449 (99.8)	43 (14.0)	0 (0.0)	<0.001
Deep hemorrhage location, *n* (%)	1,030 (55.6)	271 (60.2)	128 (41.6)	631 (57.7)	<0.001
Lobar hemorrhage location, *n* (%)	226 (12.2)	55 (12.2)	65 (21.1)	106 (9.7)	<0.001
Brainstem hemorrhage, *n* (%)	168 (9.1)	33 (7.3)	33 (10.7)	102 (9.3)	0.254
Primary ventricular hemorrhage/IVH extension, *n* (%)	35 (1.9)	10 (2.2)	15 (4.9)	10 (0.9)	<0.001
Hypertension-coded status, *n* (%)	1,509 (81.5)	416 (92.4)	0 (0.0)	1,093 (100.0)	<0.001
Diabetes, *n* (%)	131 (7.1)	37 (8.2)	8 (2.6)	86 (7.9)	0.003
CKD/renal failure, *n* (%)	247 (13.3)	63 (14.0)	23 (7.5)	161 (14.7)	0.004
Secondary-diagnosis count, median [IQR]	8 [6–12]	9 [7–13]	7 [5–11]	8 [6–12]	<0.001
ICU/neurocritical department, *n* (%)	787 (42.5)	259 (57.6)	105 (34.1)	423 (38.7)	<0.001

Clustering inputs were age, admission source, hemorrhage-location category, primary ventricular hemorrhage/IVH extension, hypertension-coded status, diabetes, and CKD/renal failure. Selected hemorrhage-location indicators are shown as binary rows. *p* values for clustering-input variables are provided only for descriptive cluster characterization and should not be interpreted as independent inferential tests. Hypertension-coded status denotes the presence of a hypertension diagnosis code in HIS; hypertension-uncoded status should not be interpreted as confirmed absence of hypertension. Sex, secondary-diagnosis count, and admission department were not used for clustering and are shown only for characterization or adjustment. IVH, intraventricular hemorrhage; CKD, chronic kidney disease; ICU, intensive care unit.

### 3.3 Unadjusted resource utilization and procedures

**Figure 1:**
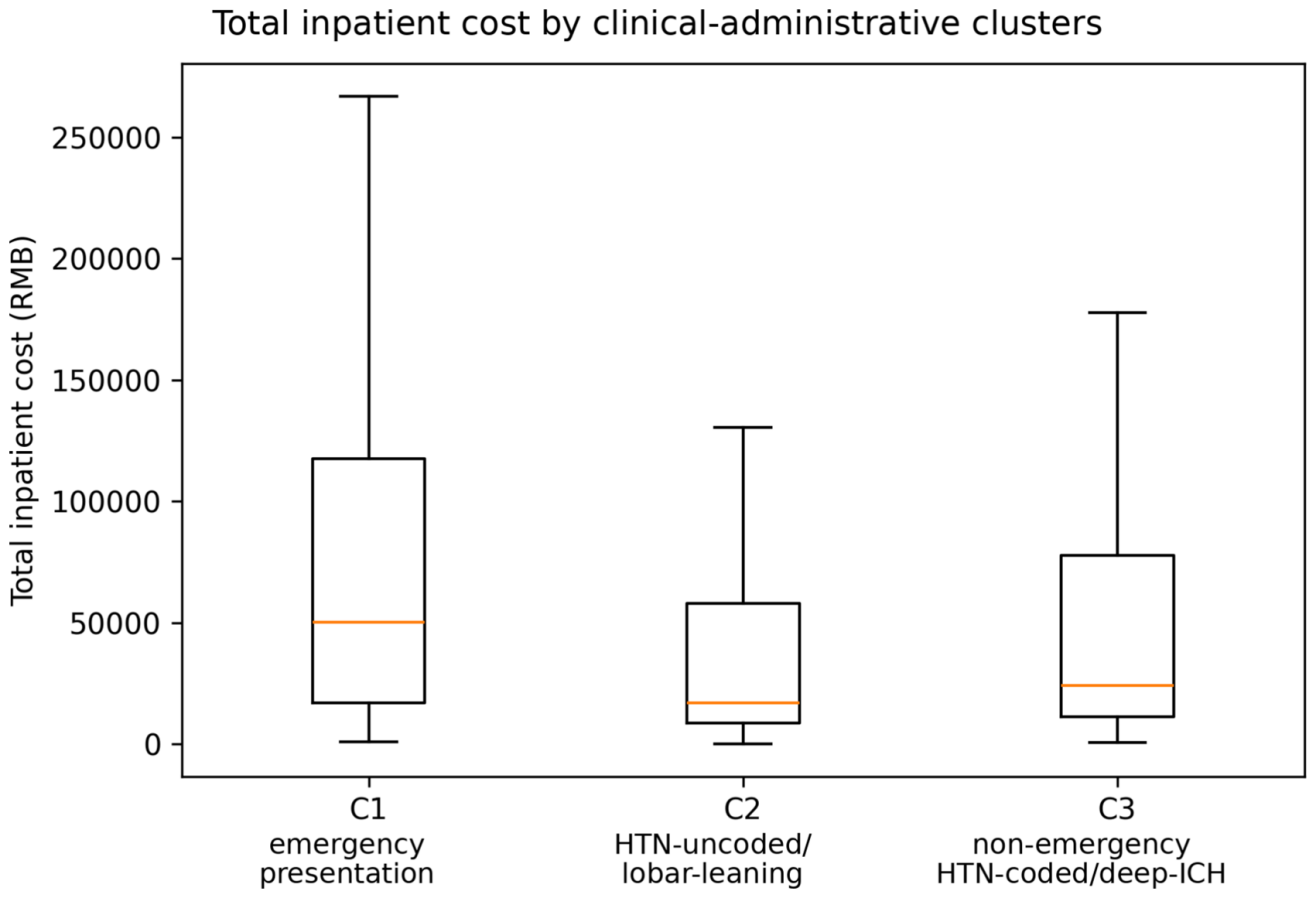
Total inpatient costs when assessed by baseline clinical-administrative clusters. Boxplots showing the medians and interquartile ranges; the outliers were omitted to improve readability. HTN denotes hypertension-coded status in abbreviated x-axis labels.

**Figure 2:**
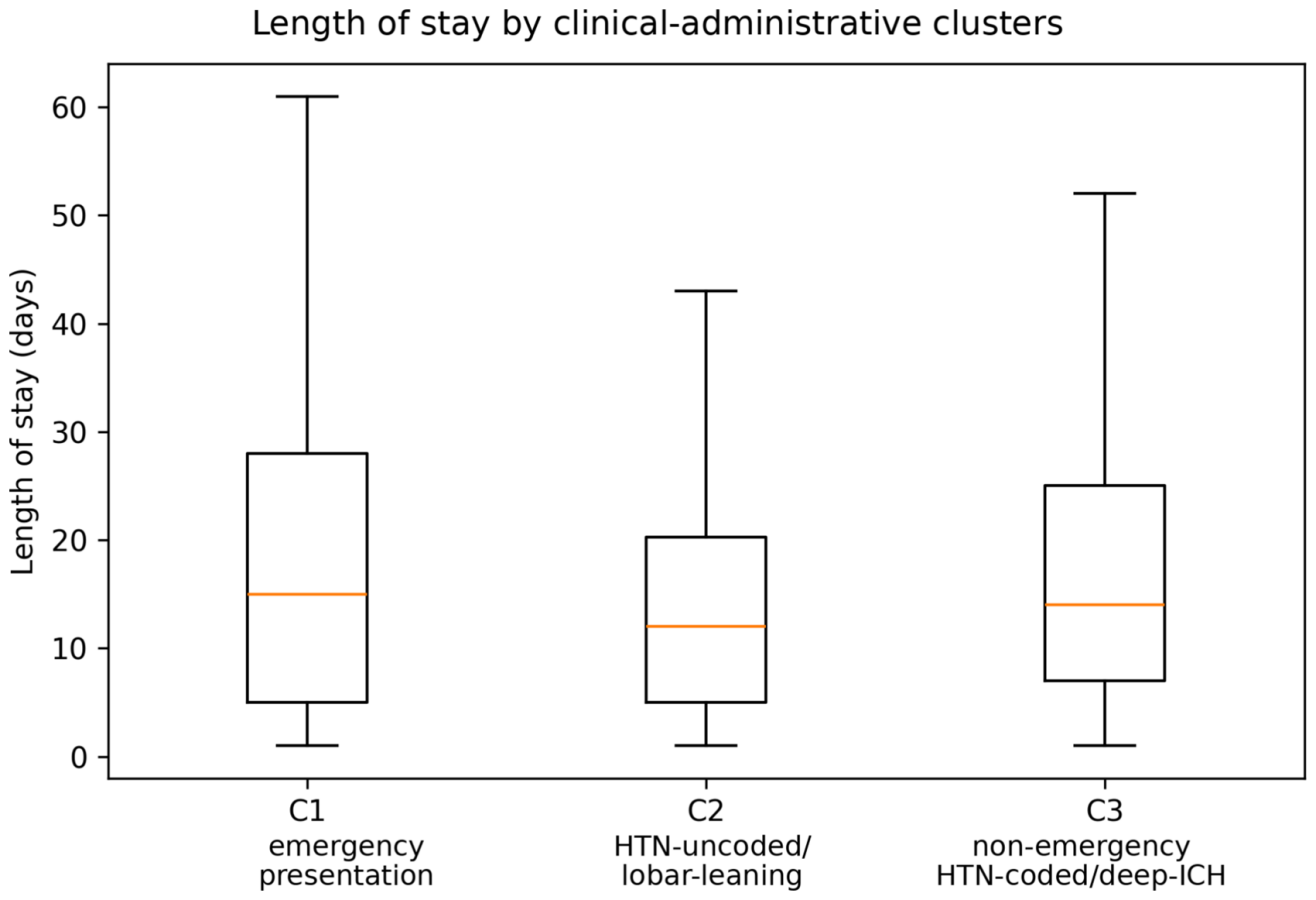
Length of stay when assessed by baseline clinical-administrative clusters. Boxplots showing the medians and interquartile ranges; the outliers were omitted to improve readability. HTN denotes hypertension-coded status in abbreviated x-axis labels.

The unadjusted utilization differed across clusters. The median total inpatient cost was highest in the emergency-presentation stratum (RMB 50,194.09) and lowest in the hypertension-uncoded/lobar-leaning stratum (RMB 16,935.15). Major neurosurgeries and tracheostomies were also most frequent in the emergency-presentation stratum, most probably because these variables were not used to form clusters, but they describe downstream utilization and care processes. Resource utilization and procedure distributions are summarized in Table 2, and the unadjusted cost and LOS distributions are shown in Figures 1, 2.

**Table 2:** Resource utilization and procedure distributions by clinical-administrative clusters.

Variable	Overall	C1 emergency-presentation	C2 hypertension-uncoded/lobar-leaning	C3 non-emergency hypertension-coded/deep-ICH	*p* value
Total inpatient cost, RMB, median [IQR]	27,452.99 [11,593.06–86,600.10]	50,194.09 [17,051.97–117,614.91]	16,935.15 [8,573.25–58,088.87]	24,133.28 [11,187.54–77,901.80]	<0.001
High cost > RMB 86,600.10, *n* (%)	463 (25.0)	158 (35.1)	56 (18.2)	249 (22.8)	<0.001
LOS, days, median [IQR]	14 [6–25]	15 [5–28]	12 [5–20]	14 [7–25]	0.004
Prolonged LOS > 25 days, *n* (%)	440 (23.8)	127 (28.2)	54 (17.5)	259 (23.7)	0.003
Any surgery, *n* (%)	1,386 (74.9)	393 (87.3)	200 (64.9)	793 (72.6)	<0.001
Major neurosurgery, *n* (%)	743 (40.1)	258 (57.3)	87 (28.2)	398 (36.4)	<0.001
Tracheostomy, *n* (%)	238 (12.9)	71 (15.8)	24 (7.8)	143 (13.1)	0.005
CVC/PICC, *n* (%)	234 (12.6)	75 (16.7)	38 (12.3)	121 (11.1)	0.011

*p* values are from Kruskal–Wallis tests for cost and LOS and Pearson Chi-square tests for categorical outcomes. LOS, length of stay; CVC, central venous catheter; PICC, peripherally inserted central catheter.

### 3.4 Multivariable outcome models

Pre-model checks supported the selected model families and all costs were positive and right-skewed (skewness, 2.22), and LOS was over-dispersed (variance-to-mean ratio, 17.81), thereby favoring negative binomial over the Poisson regression for LOS. Regression coefficients were exponentiated and are reported as cost ratios, IRRs, and ORs with 95% CIs. After covariate adjustments, the crude resource differences were attenuated. In base models adjusted for sex, payment method, admission department group, and secondary-diagnosis count, the emergency-presentation stratum was not independently associated with total cost, LOS, or prolonged LOS compared with the non-emergency hypertension-coded/deep-ICH stratum. Extended models that included complications and procedure indicators further attenuated cost differences and showed no positive independent association between the cluster membership and prolonged LOS. The inverse LOS association for Cluster 1 in the extended model (IRR, 0.90; 95% CI, 0.83–0.98; *p* = 0.010) was interpreted cautiously because this may have reflected discharge or transfer practices, mortality-related truncation, care-limitation decisions, or adjustment for in-hospital variables. Overall, the observed utilization variation was attenuated after adjustment for available administrative severity and care-process proxies. The model outputs are summarized in Table 3.

**Table 3:** Adjusted associations of clinical-administrative clusters with total inpatient cost and length-of-stay outcomes.

Model	Comparison	Cost ratio (Gamma GLM)	LOS IRR (negative binomial)	Prolonged LOS OR (logistic)
Base adjustment	C1 vs. C3	1.10 (0.99–1.24); *p* = 0.080	0.95 (0.86–1.05); *p* = 0.340	0.85 (0.64–1.14); *p* = 0.280
	C2 vs. C3	0.94 (0.83–1.06); *p* = 0.320	0.97 (0.88–1.08); *p* = 0.630	0.84 (0.58–1.23); *p* = 0.380
Extended adjustment	C1 vs. C3	0.95 (0.89–1.01); *p* = 0.110	0.90 (0.83–0.98); p = 0.010	0.78 (0.56–1.09); *p* = 0.140
	C2 vs. C3	1.01 (0.93–1.09); *p* = 0.830	1.01 (0.92–1.11); *p* = 0.770	1.06 (0.69–1.61); *p* = 0.800

Cluster 3 was the reference group. Base models adjusted for sex, payment method, admission department group, and secondary-diagnosis count. Extended models additionally adjusted for pneumonia, respiratory failure, UTI, DVT/PE, sepsis, any surgery, major neurosurgery, tracheostomy, feeding tube/gastrostomy, and CVC/PICC. Extended models were used to assess attenuation after accounting for available administrative severity and care-process proxies and should not be interpreted as causal mediation models. The inverse LOS association for Cluster 1 in the extended model should be interpreted cautiously because procedure and complication variables occur during hospitalization and may be related to discharge, transfer, mortality, or care-limitation effects. UTI, urinary tract infection; DVT/PE, deep venous thrombosis/pulmonary embolism; CVC, central venous catheter; PICC, peripherally inserted central catheter.

### 3.5 Association-rule mining results

**Figure 3:**
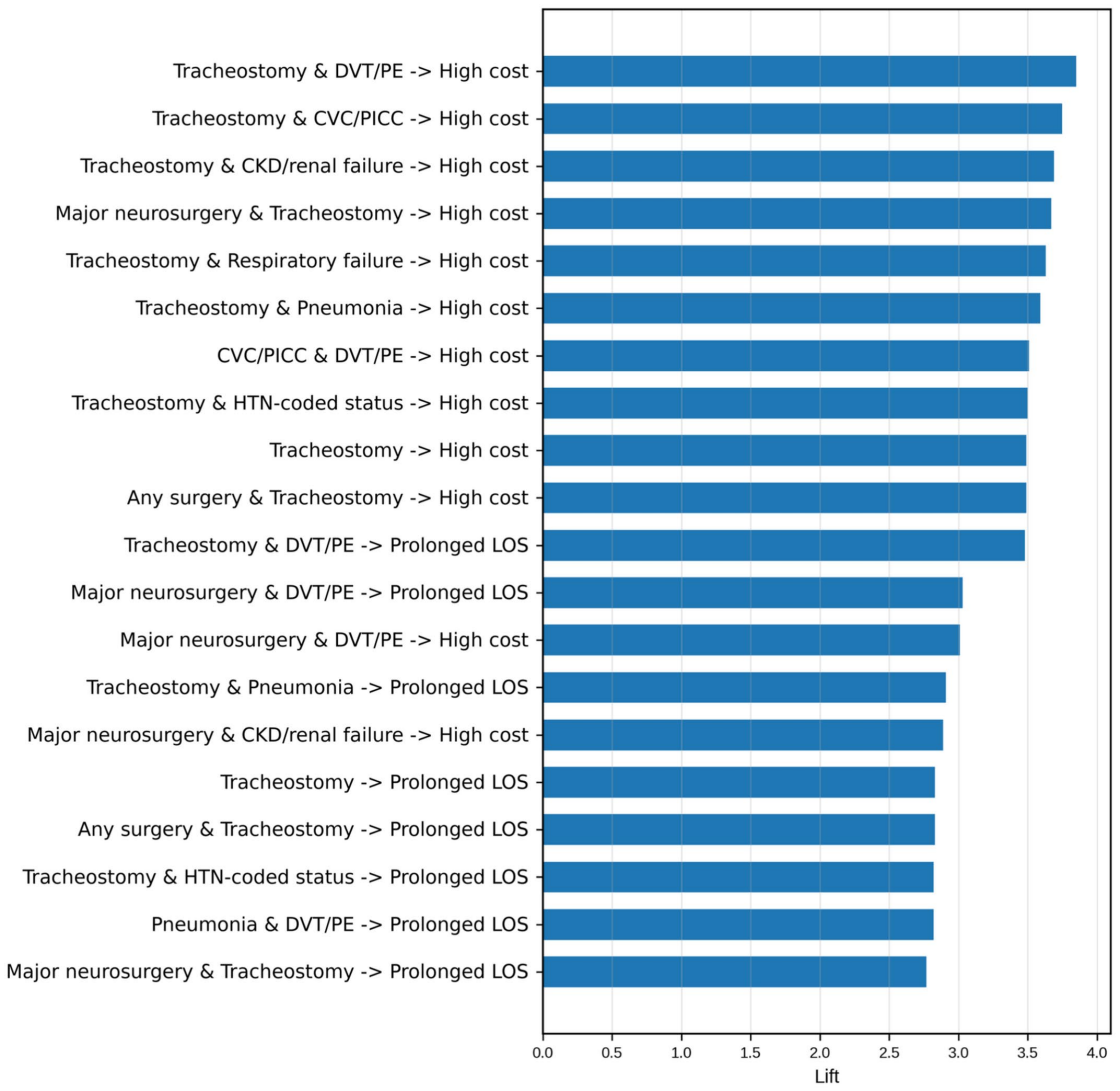
The top association rules ranked by lift for high costs and prolonged LOS. The rules met the prespecified criteria of support ≥0.02 and confidence ≥0.60. Antecedent items included selected chronic comorbidity, procedure, and acute in-hospital complication indicators; itemsets were limited to one or two items. High costs and prolonged LOS were specified only as consequents. HTN denotes hypertension-coded status.

Association-rule mining identified high-lift comorbidity/procedure/complication co-occurrence patterns associated with high cost and prolonged LOS. The strongest rules were dominated by tracheostomy-centered itemsets. Tracheostomy combined with DVT/PE had a support of 4.2%, confidence of 96.3%, and lift of 3.85 for high cost. Tracheostomy combined with CVC/PICC, CKD/renal failure, major neurosurgery, respiratory failure, or pneumonia also showed high lift for high cost. Several tracheostomy-centered combinations were associated with prolonged LOS. These rules identified where high utilization was concentrated within care pathways but did not demonstrate that a procedure caused higher cost or longer stay. The top-ranked rules are shown in Figure 3 and Supplementary Table S6.

## 4 Discussion

### 4.1 Main findings and pathway interpretation

This study combined clinical-administrative clustering with association-rule mining to characterize hospital resource-use patterns among spontaneous ICH hospitalizations. Three interpretable strata were identified including an emergency-presentation, a hypertension-uncoded/lobar-leaning, and a non-emergency hypertension-coded/deep-ICH one. The main contribution is not the self-evident observation that intensive procedures or complications are associated with higher utilization. Rather, it is the pathway-oriented organization of ICH hospitalizations into baseline clinical-administrative strata and downstream rule families, which may help distinguish like-for-like benchmarking of comparable episodes from escalation signals within the care delivery provided. Since total cost, LOS, procedures, discharge disposition, departments, and acute in-hospital complications were excluded from cluster formation, the framework reduced outcome leakage and improved the interpretability for hospital benchmarking.

Crude summaries showed higher cost, more intensive procedures, and longer LOS in the emergency-presentation stratum. This pattern is consistent with previously published ICH resource-utilization studies that operative treatment, multimorbidity, complications, and intensive care requirements contributed to higher expenditures or longer hospitalization periods (3–5). After adjustments for available administrative severity and care-process proxies, including care setting, secondary-diagnosis burden, procedures, and complications, these differences were attenuated. This contrast between crude and adjusted findings is clinically important as an emergency-presentation stratum may support an early multidisciplinary pathway review, including emergency triage, neurosurgical decision-making, neurocritical monitoring, airway planning, complication prevention, rehabilitation readiness, and discharge planning, rather than being interpreted as an intrinsically high-cost biological subtype. The clusters should therefore be used as practical clinical-administrative strata for like-for-like comparisons of similar care episodes, consistent with a structure-process-outcome view of healthcare evaluations (7).

Association-rule mining added a complementary perspective by identifying recurrent comorbidity/procedure/complication co-occurrence patterns associated with high cost and prolonged LOS. The strongest rules were dominated by tracheostomy-centered itemsets, particularly combinations involving DVT/PE, pneumonia, CVC/PICC, CKD/renal failure, respiratory failure, and major neurosurgery. These patterns are clinically plausible because they reflect airway support, invasive monitoring or vascular access, neurosurgical intervention, comorbidity burden, and complication burden. Prior neurocritical and critical care literature reports link tracheostomy, ventilator-associated pneumonia, central-line complications, and venous thromboembolism prevention, with resource use and care pathways (17–23). In this cohort, tracheostomy should be interpreted as an escalation marker within high-intensity care pathways rather than as proof that tracheostomy itself caused higher cost or a longer hospital stay. The timing of decisions after severe stroke episodes remain context dependent. Compared with studies focused on individual procedures or complications, the rule families in this study show how these elements co-occur within the same ICH hospitalization episode, providing practical triggers for pathway review rather than causal targets. The rules can help identify recurrent escalation points for quality-improvement planning, although temporal relationships should be evaluated in future longitudinal analytical studies.

These association-rule mining findings and adjusted models may support pathway-specific benchmarking, consistent with value-based healthcare and Triple Aim frameworks that consider outcomes, experience, and resource use together (24, 25). The HIS dataset did not include hematoma volume, Glasgow Coma Scale (GCS), ICH score, National Institutes of Health Stroke Scale (NIHSS), detailed neurological status, mechanical ventilation duration, intensive care unit (ICU) duration, or care-limitation decisions. Admission source, hemorrhage location, intraventricular involvement, and chronic comorbidities are therefore incomplete severity proxies, and granular clinical severity measures remain central to ICH risk stratification and quality evaluations (26–28).

These findings are also relevant under China’s DRG/DIP-based payment reform. National policy has promoted broader DRG/DIP implementation to standardize payment and improve efficiency for comparable inpatient episodes (29). However, for ICH cases, nominally comparable payment groups may still encompass heterogeneity in admission route, hemorrhage profile, ICU/neurocritical care use, complications, coding practices, discharge pathways, and step-down or rehabilitation availability. The adjusted models reinforce the need for risk-aware, pathway-specific benchmarking rather than unadjusted cost or LOS targets. Empirical evaluations of DRG/DIP-style payment reforms suggest that effects on LOS, costs, and quality can vary across settings and disease groups (30, 31). Because ICU duration is a major determinant of hospital costs in mechanically ventilated and critically ill patients, ICU-related LOS should be interpreted in the context of the overall care pathway (32, 33).

From a resource-management perspective, our findings also support pathway-aware rather than blunt utilization targets. Evidence syntheses suggest that structured clinical pathways may reduce in-hospital complications, LOS, and hospital costs or charges, while transitional-care and discharge-planning interventions can reduce some downstream utilization outcomes, although LOS effects vary by population and intervention design (34–36). For ICH services, episodes in the emergency-presentation stratum may warrant early review of escalation nodes, including neurocritical monitoring, airway/ventilation planning, pneumonia prevention, catheter practices, thromboprophylaxis, rehabilitation readiness, and discharge planning, whereas persistent LOS signals should be interpreted with respect to transfer capacity, rehabilitation access, and discharge barriers.

This work also contributes to the growing use of interpretable data-driven methods in stroke care. Recent EHR-based studies, including the interpretable machine-learning approach used by Lv et al. for predicting 30-day readmissions after stroke episodes, highlight the importance of transparent models that provide clinically interpretable risk factors (6). The present study is not a supervised prediction model, but rather, it uses clustering and association rules to describe administrative strata and co-occurrence structures for pathway review.

### 4.2 Limitations

Firstly, this was a retrospective single-center study using administrative HIS data. The hospitalization episode was the unit of analysis, and repeated admissions by the same patient could not be fully accounted for if patient-level linkage was unavailable. Complete-case exclusion may also have introduced selection bias if missingness was related to clinical severity or administrative coding. External validation is required before these clinical-administrative strata can be used as generalizable benchmarks. Secondly, key clinical severity variables, including hematoma volume, GCS, ICH score, NIHSS, mechanical ventilation duration, ICU duration, and functional outcome, were unavailable. These clinical measures are central to ICH risk stratification and guideline-based evaluations (26–28). Admission source, hemorrhage location, intraventricular involvement, and chronic comorbidities are therefore imperfect severity proxies.

Thirdly, secondary-diagnosis count, complications, and procedure indicators were captured from discharge-coded administrative data and may be affected by coding practices and LOS. Accordingly, these variables were handled as descriptive or adjustment variables and were interpreted cautiously in adjusted models. Discharge disposition was available only as an end-of-hospitalization administrative field. Therefore, the present analysis cannot determine whether residual LOS differences reflect clinical recovery, discharge against medical advice, death-related truncation, transfer practices, or step-down availability. Fourthly, the gap statistics did not identify a unique optimum. *K* = 3 was selected based on the combined evidence rather than a single metric and should therefore be considered a transparent and parsimonious working solution requiring external validation. Finally, association-rule mining identifies high-lift co-occurrence patterns but does not encode temporality or causation. The high-cost and prolonged-LOS thresholds were cohort-specific upper quartiles and should not be interpreted as universal management cutoffs.

### 4.3 Future directions

Future studies should externally validate these clinical-administrative strata in multicenter and multi-region cohorts, integrate imaging data, neurological scores, ventilation duration, ICU duration, discharge pathways, and functional outcomes, and evaluate whether cluster-informed, pathway-aware benchmarking improves resource management under DRG/DIP-oriented payment reform. Temporal pathway or sequence analyses could also test whether rule families, including tracheostomy-centered escalation signatures, accompanied procedures, or follow-up high-utilization episodes, could affect the results obtained.

## 5 Conclusion

This study used clinical-administrative clustering and association-rule mining to characterize hospital resource-use patterns among ICH hospitalizations. The *K* = 3 solution identified three interpretable strata related to presentation route, diagnosis-derived hemorrhage profile, and hypertension-coded status. Crude differences in hospital expenses and LOS were attenuated after adjustment for available administrative severity and care-process proxies. Tracheostomy-centered association rules highlighted recurrent comorbidity/procedure/complication co-occurrence patterns associated with high costs and prolonged LOS. These findings support pathway-specific benchmarking and resource-management strategies under DRG/DIP-oriented hospital management while underscoring the need for validation using granular clinical severity data and functional outcomes.

## Data Availability

The original contributions presented in the study are included in the article/Supplementary material, further inquiries can be directed to the corresponding author.
