# Supplementary material for: Combined clustering and association-rule analysis for hospital costs and length of stay in spontaneous intracerebral hemorrhage hospitalizations
Source: Front Public Health. 2026 Jun 19;14:1828871. doi: 10.3389/fpubh.2026.1828871 (PMC13328334; doi:10.3389/fpubh.2026.1828871)
Supplement: Supplementary file 1 [file Table_1.docx]

**Supplementary Materials**

# Supplementary Tables

**Supplementary Table S1. The variables included in and excluded from the clustering model.**

| **Status** | **Variable(s)** | **Rationale** |
| --- | --- | --- |
| Included in clustering | Age (years) | Baseline demographic variable; range-scaled within the cohort for Gower dissimilarity. |
| Included in clustering | Admission source (emergency vs non-emergency) | Proxy for admission route/urgency; coded from the HIS admission-source field. |
| Included in clustering | Hemorrhage-location category | Derived from the primary diagnosis name; categories were deep, lobar, brainstem, cerebellar, primary intraventricular, and other/unspecified. |
| Included in clustering | Primary ventricular hemorrhage/IVH extension | Binary diagnosis-derived marker of ventricular involvement (1 = present, 0 = absent). |
| Included in clustering | Hypertension-coded status, diabetes, CKD/renal failure | Chronic comorbidity indicators captured from diagnosis fields (1 = present, 0 = absent). Absence of a hypertension code denotes hypertension-uncoded status, not confirmed normotension. |
| Excluded from clustering | Total inpatient cost, LOS days, high cost, prolonged LOS | Direct utilization outcomes; exclusion prevents outcome leakage. |
| Excluded from clustering | Cost-composition ratios | Nursing, laboratory, imaging, surgery, anesthesia, rehabilitation/therapy, and traditional Chinese medicine (TCM) cost ratios were excluded because they directly encode resource-allocation patterns. |
| Excluded from clustering | Procedure indicators | Any surgery, major neurosurgery, tracheostomy, feeding tube/gastrostomy, and CVC/PICC were excluded because they represent downstream treatment intensity. |
| Excluded from clustering | Acute complication indicators | Pneumonia, respiratory failure, UTI, DVT/PE, and sepsis were treated as downstream process/severity markers, not clustering inputs. |
| Excluded from clustering | Admission/discharge department and discharge disposition | These variables were excluded from clustering to reduce institutional pathway and outcome endogeneity; admission department was retained for adjusted models. |
| Retained for description/adjustment | Sex, payment method, admission department, secondary-diagnosis count, procedures, complications | Used for characterization, model adjustment, attenuation analysis, and association-rule mining. |

Notes: HIS, hospital information system; IVH, intraventricular hemorrhage; CKD, chronic kidney disease; LOS, length of stay; TCM, traditional Chinese medicine; CVC, central venous catheter; PICC, peripherally inserted central catheter; UTI, urinary tract infection; DVT/PE, deep vein thrombosis/pulmonary embolism.

**Supplementary Table S2. The operational variable definitions and coding used in this study.**

| **Variable/construct** | **Definition and coding** | **Analysis role** |
| --- | --- | --- |
| Study unit and eligibility | Hospitalization episode with a primary HIS diagnosis compatible with spontaneous ICH during the study period; non-spontaneous hemorrhage and records with missing prespecified analysis variables were excluded. | Defines the analytic cohort and complete-case dataset for clustering, modeling, and rule mining. |
| Age | Continuous years at admission; range-scaled within the cohort for Gower dissimilarity. | Clustering input. |
| Admission source | Emergency versus non-emergency, coded from the HIS admission-source field. | Clustering input and presentation-route descriptor. |
| Hemorrhage-location category | Primary diagnosis-derived category: deep, lobar, brainstem, cerebellar, primary intraventricular, or other/unspecified. Deep and lobar mappings are defined in the Methods. | Clustering input. |
| Primary ventricular hemorrhage/IVH extension | 1 = primary intraventricular hemorrhage or ventricular extension recorded; 0 = not recorded. | Clustering input. |
| Hypertension-coded status | 1 = hypertension diagnosis code/name recorded; 0 = hypertension uncoded. The 0 category does not imply confirmed normotension. | Clustering input; antecedent item in association-rule mining. |
| Diabetes and CKD/renal failure | Each was coded as 1 = diagnosis code/name recorded and 0 = not recorded. | Clustering inputs; selected antecedent items in association-rule mining. |
| Secondary-diagnosis count | Number of non-primary discharge diagnosis entries recorded in the HIS. | Administrative severity/comorbidity-burden covariate. |
| Payment method and admission department group | Nominal HIS-derived administrative variables; admission department included ICU/neurocritical versus other department groups. | Adjustment covariates and descriptive variables; not clustering inputs. |
| Total inpatient cost and high cost | Total cost in RMB. High cost was coded as 1 when total cost exceeded the cohort upper quartile (> RMB 86,600.10) and as 0 otherwise. | Continuous cost was the regression outcome; high cost was a descriptive indicator and rule-mining consequent. |
| LOS and prolonged LOS | LOS in days. Prolonged LOS was coded as 1 when LOS exceeded the cohort upper quartile (> 25 days) and as 0 otherwise. | LOS was modeled as a count-like outcome; prolonged LOS was a binary outcome and rule-mining consequent. |
| Procedures | Any surgery, major neurosurgery, tracheostomy, feeding tube/gastrostomy, and CVC/PICC were coded as 1 = present and 0 = absent from structured procedure code/name mappings. | Downstream care-process variables for description, extended adjustment, and association-rule antecedents; not clustering inputs. |
| Acute in-hospital complications | Pneumonia, respiratory failure, UTI, DVT/PE, and sepsis were coded as 1 = present and 0 = absent from discharge diagnosis code/name mappings. | Downstream process/severity variables for description, extended adjustment, and association-rule antecedents; not clustering inputs. |

Notes: HIS, hospital information system; ICH, intracerebral hemorrhage; IVH, intraventricular hemorrhage; CKD, chronic kidney disease; ICU, intensive care unit; RMB, Renminbi; LOS, length of stay; CVC, central venous catheter; PICC, peripherally inserted central catheter; UTI, urinary tract infection; DVT/PE, deep vein thrombosis/pulmonary embolism.

**Supplementary Table S3. The statistical-method rationale, assumptions, and diagnostics used in this study.**

| **Method** | **Rationale and conditions for use** | **Diagnostics/reporting** |
| --- | --- | --- |
| Gower dissimilarity and k-medoids clustering | Selected because the clustering variables included mixed continuous, categorical, and binary measures. Age was range-scaled; categorical and binary variables used matching components. K-medoids was used because it accepts arbitrary dissimilarity matrices and represents clusters by observed hospitalizations. | Candidate K values from 2 to 8 were evaluated using elbow analysis, average silhouette, permutation-based gap statistic, 70% subsampling ARI, cluster size, and clinical parsimony. |
| Cluster characterization tests | ANOVA/Welch ANOVA was reserved for approximately symmetric continuous variables; Kruskal-Wallis tests were used for skewed continuous variables. Pearson chi-square tests were used for categorical variables, with Fisher's exact tests planned for sparse expected cells. | Distributions were checked using histograms, quantile-quantile plots, skewness, and variance homogeneity. Continuous variables were reported as mean ± SD or median [IQR]; categorical variables were reported as n (%). |
| Gamma GLM with log link for total cost | Selected because inpatient costs were strictly positive and right-skewed, and log-link gamma GLMs are commonly used for skewed healthcare expenditure data. | All costs were positive; cost skewness was 2.22. Results were exponentiated and reported as cost ratios with 95% CIs. |
| Negative binomial regression for LOS | Selected because LOS is count-like and the observed variance exceeded the mean, making Poisson assumptions inappropriate. | The LOS variance-to-mean ratio was 17.81. Results were exponentiated and reported as incidence rate ratios with 95% CIs. |
| Logistic regression for prolonged LOS | Selected because prolonged LOS was binary based on the prespecified cohort-specific upper-quartile threshold. | Results were reported as odds ratios with 95% CIs. Models were interpreted as associations, not causal effects. |
| Base and extended adjustment sets | Base adjustment controlled for sex, payment method, admission department group, and secondary-diagnosis count. Extended adjustment additionally included procedures and acute complications as administrative severity/care-process proxies. | Clustering-input variables were not re-entered with cluster indicators to reduce collinearity and over-adjustment. Extended models were used to assess attenuation, not causal mediation. |
| Apriori association-rule mining | Selected to describe recurrent comorbidity/procedure/complication co-occurrence patterns associated with high cost or prolonged LOS. Support and confidence thresholds were set a priori to retain clinically interpretable rules occurring in approximately 2% or more of hospitalizations while requiring that most episodes with the antecedent also met the outcome definition. | Antecedent itemsets were restricted to one or two items; consequents were high cost or prolonged LOS only. Rules required support ≥0.02 and confidence ≥0.60 and were ranked by lift. Rule outputs were interpreted descriptively, not causally. |
| Statistical significance and software | Two-sided tests used p < 0.05. Descriptive comparisons and regression models used SPSSAU version 26.0; data checking, Gower distance calculation, k-medoids clustering, K-validation summaries, association-rule summaries, model-output compilation, and figures used Python 3.11 packages. | Software and thresholds are reported to support reproducibility. Cluster comparisons and rule mining were exploratory/descriptive; p values and rule rankings were interpreted without multiplicity-adjusted confirmatory claims. |

Notes: GLM, generalized linear model; ANOVA, analysis of variance; LOS, length of stay; ARI, adjusted Rand index; SD, standard deviation; IQR, interquartile range; CI, confidence interval.

**Supplementary Table S4. The full K-validation metrics used in this study.**

| **K** | **Within-cluster dissimilarity** | **Relative W decrease (%)** | **Average silhouette** | **Gap statistic (SE)** | **Subsampling ARI mean (SD)** | **Cluster sizes** |
| --- | --- | --- | --- | --- | --- | --- |
| 2 | 258.9 | — | 0.408 | 0.020 (0.036) | 0.788 (0.403) | 493, 1,358 |
| 3 | 220.9 | 14.7 | 0.440 | 0.015 (0.017) | 0.702 (0.212) | 450, 308, 1,093 |
| 4 | 195.2 | 11.6 | 0.359 | 0.068 (0.047) | 0.602 (0.159) | 423, 813, 328, 287 |
| 5 | 175.2 | 10.3 | 0.371 | 0.076 (0.044) | 0.619 (0.156) | 398, 685, 279, 296, 193 |
| 6 | 162.7 | 7.1 | 0.382 | 0.079 (0.041) | 0.606 (0.110) | 390, 181, 572, 275, 263, 170 |
| 7 | 149.5 | 8.2 | 0.403 | 0.097 (0.047) | 0.610 (0.076) | 361, 151, 551, 207, 189, 168, 224 |
| 8 | 141.9 | 5.1 | 0.436 | 0.085 (0.047) | 0.648 (0.090) | 624, 207, 165, 146, 333, 170, 138, 68 |

Notes: K, number of clusters; W, within-cluster dissimilarity; SE, standard error; ARI, adjusted Rand index; SD, standard deviation. Gap statistics were calculated using 20 independently permuted reference datasets; stability was evaluated using 40 random 70% subsamples and summarized as mean ARI (SD). The gap statistic did not identify a unique optimum, so K was selected by jointly considering average silhouette coefficient, elbow improvement, subsampling stability, cluster size, clinical interpretability, and parsimony. K = 3 was retained as the parsimonious working solution.

**Supplementary Table S5. The cluster-specific silhouette values for the selected K = 3 solutions.**

| **Cluster** | **n** | **Mean silhouette** | **Median silhouette** | **IQR** | **Minimum** |
| --- | --- | --- | --- | --- | --- |
| Cluster 1 | 450 | 0.401 | 0.411 | 0.361-0.516 | -0.105 |
| Cluster 2 | 308 | 0.346 | 0.410 | 0.309-0.426 | -0.068 |
| Cluster 3 | 1,093 | 0.482 | 0.470 | 0.421-0.564 | 0.247 |

Notes: Silhouette values were calculated from the Gower dissimilarity matrix. IQR, interquartile range.

**Supplementary Table S6. The top 20 association rules ranked by lift.**

| **Antecedent itemset** | **Outcome** | **Support (%)** | **Confidence (%)** | **Lift** | **Rule items** |
| --- | --- | --- | --- | --- | --- |
| Tracheostomy & DVT/PE | High cost | 4.2 | 96.3 | 3.85 | 3 |
| Tracheostomy & CVC/PICC | High cost | 3.2 | 93.8 | 3.75 | 3 |
| Tracheostomy & CKD/renal failure | High cost | 2.6 | 92.3 | 3.69 | 3 |
| Major neurosurgery & Tracheostomy | High cost | 10.2 | 91.7 | 3.67 | 3 |
| Tracheostomy & Respiratory failure | High cost | 2.1 | 90.7 | 3.63 | 3 |
| Tracheostomy & Pneumonia | High cost | 10.5 | 89.9 | 3.59 | 3 |
| CVC/PICC & DVT/PE | High cost | 2.3 | 87.8 | 3.51 | 3 |
| Tracheostomy & HTN-coded status | High cost | 9.9 | 87.6 | 3.50 | 3 |
| Tracheostomy | High cost | 11.2 | 87.4 | 3.49 | 2 |
| Any surgery & Tracheostomy | High cost | 11.2 | 87.4 | 3.49 | 3 |
| Tracheostomy & DVT/PE | Prolonged LOS | 3.6 | 82.7 | 3.48 | 3 |
| Major neurosurgery & DVT/PE | Prolonged LOS | 8.0 | 72.0 | 3.03 | 3 |
| Major neurosurgery & DVT/PE | High cost | 8.4 | 75.4 | 3.01 | 3 |
| Tracheostomy & Pneumonia | Prolonged LOS | 8.1 | 69.1 | 2.91 | 3 |
| Major neurosurgery & CKD/renal failure | High cost | 4.4 | 72.3 | 2.89 | 3 |
| Tracheostomy | Prolonged LOS | 8.6 | 67.2 | 2.83 | 2 |
| Any surgery & Tracheostomy | Prolonged LOS | 8.6 | 67.2 | 2.83 | 3 |
| Tracheostomy & HTN-coded status | Prolonged LOS | 7.6 | 67.1 | 2.82 | 3 |
| Pneumonia & DVT/PE | Prolonged LOS | 9.1 | 66.9 | 2.82 | 3 |
| Major neurosurgery & Tracheostomy | Prolonged LOS | 7.3 | 65.9 | 2.77 | 3 |

Notes: Rules were generated using support ≥0.02 and confidence ≥0.60. High cost and prolonged LOS were defined using cohort-specific upper-quartile thresholds. Antecedent items included selected chronic comorbidity, procedure, and acute in-hospital complication indicators; antecedent itemsets were limited to one or two items. The rules describe co-occurrence patterns and should not be interpreted as evidence of temporality or causation. HTN, hypertension; CKD, chronic kidney disease; CVC, central venous catheter; PICC, peripherally inserted central catheter; DVT/PE, deep vein thrombosis/pulmonary embolism; LOS, length of stay. The rule-items column counts the antecedent item(s) plus the consequent.

# Supplementary Figures


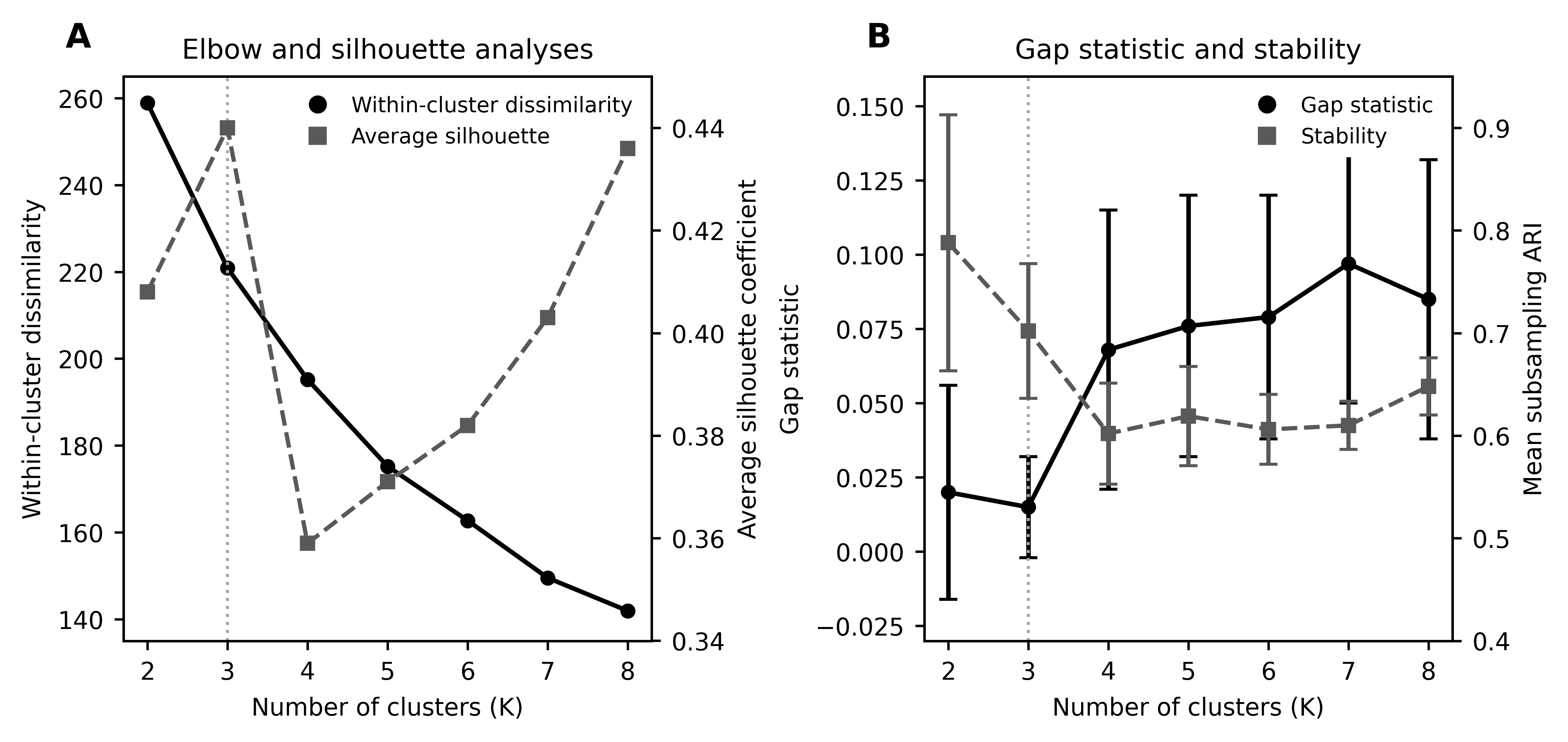


**Supplementary Figure S1.** K-validation metrics across candidate numbers of clusters (K). (A) Elbow analysis and average silhouette coefficients. (B) Permutation-based gap statistics and subsampling stability. Error bars indicate ±1 standard error for the gap statistic estimates and 95% confidence intervals for the mean adjusted Rand index from subsampling.
